# Pet roundworms and hookworms: A continuing need for global worming

**DOI:** 10.1186/1756-3305-5-91

**Published:** 2012-05-10

**Authors:** Donato Traversa

**Affiliations:** 1 Department of Comparative Biomedical Sciences, University of Teramo, Teramo, Italy

## Abstract

Ascarids and ancylostomatids are the most important parasites affecting dogs and cats worldwide, in terms of diffusion and risk for animal and human health. Different misconceptions have led the general public and pet owners to minimize the importance of these intestinal worms. A low grade of interest is also registered among veterinary professions, although there is a significant merit in keeping our guard up against these parasites. This article reviews current knowledge of ascarids and ancylostomatids, with a special focus on pathogenicity, epidemiology and control methods in veterinary and human medicine.

## Review

### Background

The relationship between human beings and domesticated small animals began about 15.000 years ago [1]. Such association has led to the dispersion of pets all over the World, along with the spread of their pathogens. Some of them are common and zoonotic: as a consequence, there is a continuing interest on their sanitary impact, and on prevention and control methods. In the past few years the attention of the Scientific Community has been attracted by feline and canine extra-intestinal parasitic nematodes, which are emerging in several countries and spreading into regions previously free from these parasites. Indeed, global climate change is influencing the ecology of helminths with multiple hosts and different transmission routes. As key examples, this is the case of the insect-borne filariae and eyeworm [2-4] and of the snail-borne lungworms *Aelurostrongylus abstrusus* and *Angiostrongylus vasorum*[3,5]. This new concern has caused the misconception that intestinal worms of cats and dogs do not deserve a high-standard level of attention anymore, especially because the routine use of certain anthelmintics is believed to have reduced their diffusion and impact on animal health and welfare [6]. Indeed, the use of broad spectrum drugs, which are sold (often over-the-counter) in a plethora of formulations, carries the risk that leads the general public to minimize the importance of the “common intestinal worms” and to erode the importance of the veterinarian in controlling parasites of veterinary and human impact. This low-grade of interest and attention is crucial if one considers that several pet intestinal nematodes are zoonotic and endemic globally, and the spread of these parasites may be favoured by current climate changes. In fact, these parasites have periods of development and survival in the environment, which are often at the basis of transmission routes in important sapro-zoonoses.

Different species of ascarids (commonly known as “roundworms”) and ancylostomatids (commonly known as “hookworms”) may affect the small intestine of dogs and cats [7]. Actually, they remain the most important parasites affecting companion animals worldwide and maintain the primacy in terms of dispersion and risk for animal and human health. This is of particular importance also because some driving forces are nowadays favouring their spread, e.g. the increase of wild fox populations in sub-urban and urban areas. For example, wild foxes may act as reservoirs and amplifiers of canine ascarids, thus they re-enforce environmental contamination and risk of infection [8].

There is, in turn, a significant merit in keeping our guard up against these nematodes even when other parasites are attracting attention and interest. Therefore, the aim of this article is to review the most important features of roundworms and hookworms affecting companion animals, along with critical and focused appraisals on the importance of their pathogenicity, epidemiology and control methods in veterinary and human medicine.

### Intestinal nematodes are complex and interesting

Pets can be parasitized by different nematodes, intestinal roundworms, hookworms and whipworms being the most common. Main aspects of trichuroid whipworms (i.e. *Trichuris vulpis*) affecting the large bowel of dogs have been recently described elsewhere [9], thus this species will be excluded from this article.

*Toxocara canis* and *Toxocara cati* are the two major ascarids globally infecting dogs and cats, respectively. Both species have a complex and fascinating biological cycle, which relies on different pathways of larval migrations and transmission, depending upon mainly the source of infection and animal age.

Bitches are a major source of infection for their off-spring because, after an infection occurs in their life, they harbour somatic larvae. These resting larvae will mobilize during pregnancies and infect subsequent litters even when re-infections do not occur. Pups become infected *in utero* by the second month of gestation, which result in egg shedding after a minimum period of about two weeks after birth [10,11]. When mobilized larvae are transmitted via the lactogenic route litters can also be infected by colostrum and milk for at least 38 days after delivery [12,13]. These vertical infections occur regardless of the presence of the intestinal parasitosis in the bitch but, in general, a proportion of mobilized larvae may reach the intestine of the dam, then mature and cause a patent infection with high egg shedding lasting weeks after whelping. Bitches can be re-infected also by ingesting immature ascarids defecated by their suckling offspring. Therefore, lactation may either cause or reinforce a patent infection in bitches, which provides another source of environmental contamination and infection for puppies [13].

While *T. cati* is not transplacentally transmitted, lactogenic infection may occur in kittens during the first days of nursing [14,15]. It has been recently shown that the acute infection of the queen during a late phase of pregnancy causes the milk-borne infection in the offspring [15].

Dogs and cats of all ages can also acquire the infection by ingesting *Toxocara* embryonated eggs from the environment and eating paratenic hosts (e.g. invertebrates, ruminants, rodents, birds) harboring tissutal larvae [13,16-18].

The prepatent period for toxocarosis by *T. canis* is at minimum 4–5 weeks after ingestion of embryonated eggs or resting larvae, and 2–3 weeks for prenatal infections, while kittens start to shed *T. cati* eggs after about 7–8 weeks post infection [13,19,20].

A third roundworm, *Toxascaris leonina*, affects both dogs and cats. This species is, in general, less diffuse than *Toxocara* spp., especially because transmissions via the placenta and mammary glands do not occur. Animals become infected only by ingesting larvated eggs from the environment or tissutal larvae in paratenic hosts, e.g. rodents [13].

Pathogenesis and symptoms due to adult stages are similar to *Toxocara* spp. (see below) but the infection does not occur in animals aged less than about 2 months. Prepatency period is about 10–11 weeks [20,21].

Among the most common hookworms, *Ancylostoma caninum* and *Ancylostoma tubaeforme* are species-specific for dogs and cats respectively, while *Ancylostoma brazilienseAncylostoma ceylanicum* and *Uncinaria stenocephala* affect both species [7,17,22]. In general *A. caninumA. tubaeforme* and *U. stenocephala* are spread especially in warm countries (*Ancylostoma* spp.) and in colder areas of temperate and subarctic regions (*U. stenocephala*) in both hemispheres; the remaining hookworms are most often present in sub-tropical and tropical countries [20,23-26]. As for roundworms, hookworms have a complex biological cycle, in which different sources and ways of infection are possible. The most important infectious stage is represented by filariform larvae present in the soil, which infect a suitable host by actively penetrating the skin (especially for *Ancylostoma* spp.) and/or via the oral route (i.e. *Ancylostoma* spp., *Uncinaria* spp.) [7,13,22,27].

Nursing is a relevant source of infectious larvae of *A. caninum* for puppies. In fact, it is well established that, when infection occurs in adult dogs, a proportion of larvae invade different body regions. These resting stages survive for years and are, in turn, reactivated during oestrus and in the last 2–3 weeks of pregnancy, when they are passed via the milk to the litter for at least 3 weeks after delivery [26,28-30]. A bitch harbouring somatic larvae is infectious for three consecutive litters, although the larval output is reduced in each lactation [30-34]. Conversely, there is a scattered and conflicting body of bibliographic information on the transplacentary transmission [13,20,26,35,36]. Indeed, if *in utero* infection occurs at all, it is obfuscated by the lactogenic route and, in any case, prenatal transmission by *A. caninum* does not occur in all puppies from a litter [7,27,37-39]. It has also been reported that larvae of *A. caninum* dormant in musculature may be re-activated following factors driving stress, e.g. severe illness or corticosteroid therapies, which then reach the intestine causing patent infections in the adult dog [27]. *In utero* and lactogenic infections do not occur for *A. tubaeforme*, even though literature is scarce and the extent of milk transmission is stated to be not well known [7,17,27]. For the other canine and feline hookworms vertical infections do not appear to occur at all [7,17,39,40].

Paratenic hosts are also important in transmitting ancylostomosis in dogs and cats which prey on animals (e.g. rodents). Prepatent period for *A. caninumA. tubaeforme* and *U. stenocephala* is about 2–3 weeks [7,17,20].

In summary, there are major factors making roundworms and hookworms the most common endoparasites in pets all over the World. First of all, the possibility of puppies and kittens being infected by their dam by transmammary and/or transplacental route/s is a powerful host-finding strategy. Also, pups have daily thousands epg counts for *T. canis* and animals often shed millions of hookworm eggs for weeks, thus causing a high environmental contamination. Ascarid eggs can survive for years in extreme environmental conditions, thus are available for ingestion at any time. Infected paratenic hosts are ubiquitous, being a constant source of infection especially for cats, given their hunting instinct.

### Is age a decisive circumstance for host-finding strategies of intestinal nematodes?

There is a long-standing misconception on the age categories of dogs and cats, which can be infected. In fact, it is often thought that “intestinal worms” are only a health problem of puppies and kittens and that adult animals are, instead, resistant.

The real truth is that pets are exposed to roundworm and hookworm infections throughout the year and for all their life. Specifically, parasitic burdens, egg output and infection rates are higher in puppies and kittens but it is nowadays established that patent intestinal infections occur in dogs and cats of all ages [41-50].

Adult dogs can be re-infected by *T. canis* even when under regular control programs [46,51]. Also, they have the same susceptibility for patent infections as naïve patients when later re-exposed and even when repeatedly exposed to the parasite and having circulating antibodies *vs* ascarid surface antigens [52,53]. Patent ascaridosis may be detected in animals older than 3 years or more, and may also establish when infection occurs with a few larvated ova [11,46,54-56]. Also, nursing bitches may present heavy patent infections by about 4 weeks after delivery [27].

Analogously, the chance that a cat develops a patent intestinal infection by *T. cati* remains high throughout its life. For instance, one of the major causes of infection for adult cats is the ingestion of larvated eggs acquired from the environment by their perpetual self-grooming [27].

For its biological cycle, the infection by *T. leonina* is much more common in adult animals than in young subjects [27].

Some studies have unwaveringly indicated a significantly higher prevalence of canine hookworms in young dogs [41,44,47,57]. Nonetheless, there is evidence that prevalence of *A. caninum* in dogs <11 months of age can be significantly lower than infection rates in dogs aged 1–6 and >6 years [58] and that there is no relationship between host age and prevalence of *Ancylostoma* spp. [59]. Analogously, prevalence of *U. stenocephala* can be higher in dogs of more than 3 years of age than in puppies of less than 4 months [60]. Surveys performed in the USA have underscored high infection rates in young puppies and only slight age-related decrease of prevalence after 1–2 [47] or 7 years of age [54]. It is worth mentioning that, after resting larvae of *A. caninum* are re-activated in pregnancy, they can cause auto-infection of the dam, thus reinforcing opportunities for adult dogs to show patent ancylostomosis [13]. In general, old dogs infected by *A. caninum* usually display a prolonged prepatency and a reduced period of egg shedding, likely due to partial immunity or age resistance [13]. Given that *A. tubaeforme* is not transmitted *in utero* or via the milk, the infection can be present in cats of all ages and not only in kittens. There are studies that have shown an increasing trend of infection rate in 1–5 year old cats rather than in younger animals [42,53].

An investigation carried out in the USA on the most common canine and feline endoparasites in thousands of pets has shown that, after animals under 6 months of age (as expected), the most parasitized category of animals are patients more than 10 years old [53]. The possible explanation of such a high degree of parasitism in old animals may reside in a loss of immune response against previously experienced parasites [61,62]. Another possible reason may be a loss of compliance of pet owners, who, perhaps, become less willing to engage in chemopreventative measures in old pets [53]. Such changing approach of pet owners should be discouraged by veterinarians not only for the pathogenicity of intestinal worms, but also because there is no practical reason to consider an old animal a less effective source of infection for other pets and human beings in comparison to puppies and kittens.

### Biology and pathogenicity of intestinal nematodes: Threats for pets and humans

Virtually 100% of dogs and cats, from the cosseted and beloved pet to the stray animal, have been in contact with ascarids and ancylostomatids or, at least, are at risk of disease.

**Figure 1:**
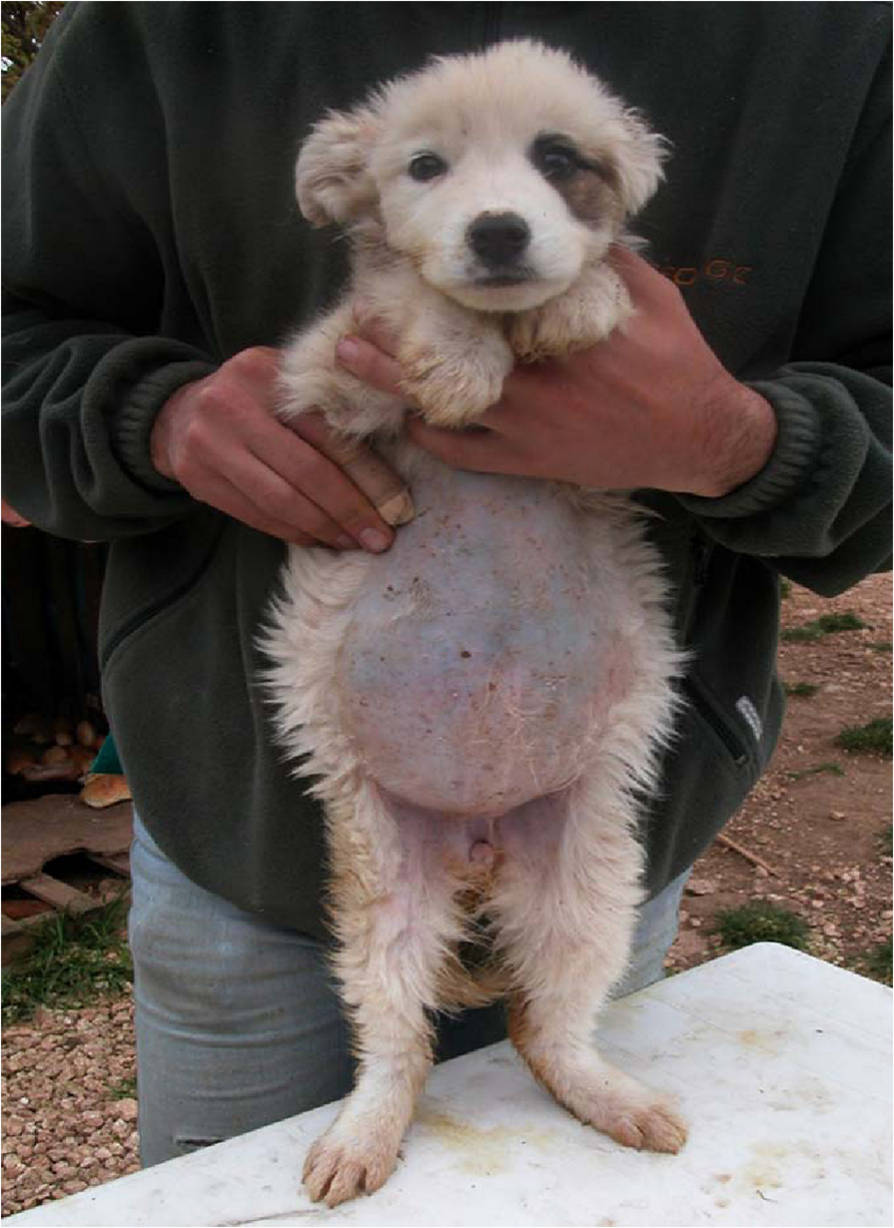
Pot belly in a roundworm-infected puppy.

**Figure 2:**
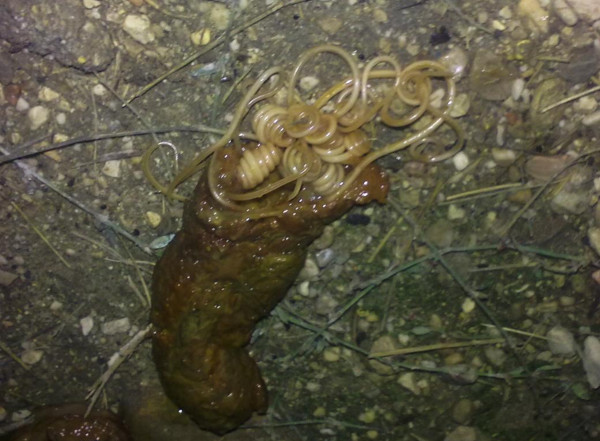
**Adults of*Toxocara canis*spontaneously expelled with the faeces by a puppy.**

Ascarids live free in the lumen of the small intestine feeding on its content. Mild infections are usually not accompanied by clinical signs either in larval migration or in patent infections. When the number of canine roundworms is moderate-high, larval migrations can cause cough, frothy nasal discharge, pneumonia and edema of the lungs. Death mostly occurs in this larval phase and especially within a few days after birth in puppies borne after a severe transplacental infection [13]. Adult roundworms in pups cause by the second-third week of age a mucoid enteritis characterized by vomiting, diarrhoea, ascites, anorexia, anaemia, unthriftiness, emaciation, poor coat, nasal discharge, and pot belly (Figure 1) due to heavy worm burden, dysbacteriosis and gas formation. In severe circumstances animals may suffer from thickened intestine, partial or total obstruction or occlusion, duodenum dilatation, peritonitis, bile and pancreatic ducts blockage, rupture of the intestine and worms at different stages expelled with the vomitus or the faeces (Figure 2); indeed, pups and kittens with heavy infections may expel a large mass of worms in vomitus [13,16,20,27], thus causing distress for the owner as the worms are large and usually alive. Nursling puppies suffer severe discomfort, whimper and shriek and, when walking or standing, present a straddle-legged posture of the hind limbs [27]. Penetration of the peritoneal cavity after gut wall perforation, with subsequent peritonitis and massive blood loss, have also been reported [63,64].

Infected kittens may show a catarrhal enteritis with variable appetite, vomiting after feeding, diarrhoea alternated with constipation, developmental disturbances, anaemia and anorexia, especially after severe infections [65,66]. In adult cats *T. cati* may cause vomiting, enlargement of the abdomen, anorexia and even gastric perforation with presence of adult parasites in the abdominal cavity [67,68].

A case of a feline infection by *T. canis* characterized by an eosinophilic granulomatous dissemination of larvae has been reported in a pyrexic cat [69].

*Toxascaris leonina* has a less dramatic pathogenicity, but in dogs it may cause pica, digestive disturbances and reduced growth, while in cats the infection can be seen as an enteritis with vomiting and diarrhoea, even bloody [17,70,71].

Hookworms have been regarded as the most faithful intestinal parasites of dogs and cats [36]. Indeed, they are responsible for developmental impairment, severe clinical signs and high death rate, especially in young subjects. These worms live anchored to the gut mucosa by their oral capsule and have a relevant blood-sucking activity. Indeed, while *A. braziliense* may present a mild pathogenic impact, the other hookworms are intensive hematophagous and cause important exsanguination. In general, ancylostomosis in pets induces a mild enteritis to a fatal hemorrhagic diarrhoea with anemia, depending upon different drivers, e.g. age of the animal, parasitic burden and species involved [16,17,20,27,29].

Juveniles of *A. caninum* burrow deeply and massively into the mucosa, thus symptoms can be severe and life-threatening especially for puppies, in which even fatal diseases may occur in pre-patency. After a milk-borne infection, pups, which have low iron reserves, are healthy in the first week of age, and then show profound blood loss and deteriorate rapidly within the second-third week after birth. Age is a crucial factor in the outcome of canine ancylostomosis, because as the animal grows, resistance increases, regardless of whether the animal has experienced one or more infections. However, further infections may be inhibited by a pre-existent hookworm populations (i.e. premunition) and, in general, symptoms in adult dogs are dependent upon state of nutrition, hematopoietic capacity, presence of stressful conditions. In general, adults with a mild parasitic burden with *A. caninum* suffer with a moderate anaemia for the capacity to which their bone marrow has to compensate, but these animals may develop a more intense microcytic hypochromic anaemia in any case. Heavy infections always present with chronic iron deficiency anaemia, poor coat, loss of weight, bloody and mucous faeces, reduced growth, circulatory collapse, lack of stamina and poor general physical condition [13,20,27,36].

Cats affected by *A. tubaeforme* suffer with enteritis, blood loss, diarrhoeic faeces, reduced weight, regenerative anemia, cachexia and even death [13,65,72].

*Uncinaria stenocephala* has a less intense haematophagous activity compared to *Ancylostoma* spp. and removes small amounts of blood; heavy infections in young animals may be characterized by mild anaemia, hypoalbuminemia, anorexia, diarrhoea and lethargy [20,27].

The majority of canine and feline roundworms and hookworms are potentially zoonotic.

Human beings can be hosts of ascarids when they inadvertently ingest embryonated eggs from the soil (i.e. sapro-zoonosis) or tissutal larvae. For instance, this happens by putting unwashed fingers into the mouth, or eating raw contaminated vegetables or meat of paratenic hosts [73]. As an example, a relatively common source of human infection is food, represented by vegetables harvested from farms using animal dung as fertilizer [18,73,74] or by raw or undercooked liver or meat from ruminants, pigs, chickens [18,75,76]. A relatively unexploited source of infection for humans is represented by embryonated ascarid eggs present on dogs’ fur [56,77]. The real proportion of pets whose fur is truly contaminated by infectious eggs and whether they are a real threat for humans still remains to be elucidated. However, direct contact with an animal infected by roundworms should not be considered hazardous for the following reasons: *Toxocara* eggs need about 2–6 weeks before infectivity is reached, they are strongly adhesive on animal’s coats and difficult to ingest, most of them are not viable, and, finally, several fur grams need to be swallowed to cause infection [78-84]. Moreover, the presence of non-canid parasitic ova on fur of dogs indicates that animals become contaminated in the environment, possibly through scent-rolling, rather than from their own defecations [56].

Once infective elements are ingested, larvae penetrate the gut wall and reach the bloodstream wandering throughout the body, i.e. eyes, heart, muscles, brain, lungs, liver. Thereby, larvae do not molt nor reach the adulthood but, however, induce severe local reactions and damage, which may lead to different syndromes (see next section).

With regard to hookworms, pets and humans suffer from a skin condition when free-living infective larvae species present in the soil, enter into the skin to reach the intestine via the bloodstream. In pets the cutaneous damage leads to different degrees of itching, erythema, vesicular or papular lesions, acanthosis, hyperkeratinization, cellular infiltrates and perivascular cuffing, especially at the interdigital skin [22,26,36]. However, infectious larvae of animal hookworms may penetrate human skin, causing local lesions, intestinal distress and even ocular/neurological signs; however, the aetiological role of each animal hookworm in causing disease in humans remains to be elucidated (see next section).

### Pet roundworms and hookworms are zoonotic

Soil-transmitted helminthoses affects more than 2 billion people worldwide [85]. The zoonotic ability of *Toxocara* spp. has been established since the 1950’s [86,87] and presently it is well known that pet ascarids cause human infections globally, as demonstrated by several surveys carried out in all corners of the World [18,88].

Indeed, *T. canis* is largely acknowledged as a major culprit of human syndromes by animal ascarids, but it is likely that some human infections are caused by *T. cati* as well [88,89].

Some infections are asymptomatic [90,91] and the degree of damage and elicited signs depend upon the tissue/s invaded, number of migrating larvae, host age and immune response. When symptoms are present, two major syndromes may occur, i.e. the so-called “visceral *larva migrans*” (VLM), encompassing important organs (mainly liver, lungs, brain) and “ocular *larva migrans*” (OLM), due to damage to eye and optic nerve; other minor syndromes, e.g. covert, neural, and atopyc toxocarosis, are also reported [88,90,91].

Children, in particular toddlers, are the most frequent subjects suffering from VLM, often with severe clinical symptoms. The high occurrence in children is due to frequent exposure to areas (e.g. sandpits, sandboxes, gardens, playgrounds) potentially contaminated by *Toxocara* eggs and to low hygiene standards [79]. Also, geophagic pica (e.g. due to iron or zinc deficiency or to behavior disorders), which may affect up to 10% of children, is another relevant risk factor [79,92].

VLM in 1–5 year old children is characterized by fever, leucocytosis, eosinophilia, hypergammaglobulinaemia, general malaise, abdominal distress and pain; when larvae infect the liver, patients can suffer from hepatomegaly, granulomatous hepatitis or even necrosis [79,93-95]. There are cases of bronchiolitis/pneumonitis with wheezing, cough and asthma-like bronchospasm, and of myocarditis, nephritis, and involvement of the central nervous system with meningoencephalitis, seizures, and neuropsychiatric signs [79,88,94,96-98].

OLM usually occurs without signs of VLM in children aged 5–10 years and in adults as well [92,99], and is characterized by impaired vision to total loss of sight, due to endophthalmitis, retinal granulomas and detachment of the macula [88,100]. Other signs are complaints of “seeing lights”, squint and glaucoma; more importantly, OLM may mimic a retinoblastoma, thus erroneously inducing *enucleatio bulbi*[99,101,102]. Hundreds of cases of unilateral blindness and of more or less severe eye damage have been calculated to occur yearly in US childhood due to OLM and dozens of unnecessary eye enucleations due to misdiagnosis with retinoblastoma are described [6,27,74].

A third condition, called “covert toxocarosis”, might be caused to long-term exposure to migrating larvae in specific target organs. In children older than toddlers, this form presents vaguely with behavioral changes, seizures and sleep alterations, cough, asthma, abdominal discomfort, headache, while in adults it may present with weakness, rash and itching, abdominal pain and breathing distress [103-108].

Finally, skin conditions, like pruritus, urticaria and different eczematous lesions, have been found in association with toxocarosis in both childhood and adults [88,109].

There are reports of juveniles of feline roundworms identified in the liver and the brain of two children from the USA and Israel [110,111]. Interestingly, there are published cases of adult *T. cati* passed from the anus or the mouth of children, but it is very likely that these cases originated by an altered behavior, i.e. taking worms from vomitus or faeces from an infected animal [112,113]. It seems that *T. leonina* does not display zoonotic potential. However, there are a couple of published reports of possible human infections by this species: a case of osteomyositis with cutaneous abscesses containing worms identified as *T. leonina* has been described in the 1960’s in the former USSR [114], while a case of ocular infection by a suspected *Toxascaris* spp. larva has been described from Africa [115].

Humans may be infected by free-living hookworm larvae when walking barefoot, when in close contact with potential contaminated soil (e.g. gardeners), or when sunbathing on beaches in risky areas. Larvae of *A. braziliense* cause the so-called “cutaneous *larva migrans*” (CLM), a dermatitis with long serpiginous and persistent tracks underneath the human skin. The role of other hookworms in causing CLM needs to be clarified. The US CDC states “CLM” to be known also as “creeping eruption”, being “a zoonotic infection with hookworm species that do not use humans as a definitive host, the most common being *A. braziliense* and *A. caninum*” [116]. Also, the CDC states that “A larger group of hookworms infecting animals can invade and parasitize humans (*A. ceylanicum*) or can penetrate the human skin (causing cutaneous *larva migrans*), but do not develop any further (*A*. *brazilienseA. caninumUncinaria stenocephala*)” [116].

Indeed, the geographic distribution of CLM overlaps that of *A. braziliense*[25,117] and interestingly, it does not occur where this species is absent, e.g. in Mexico, West US coasts and Australia [24,26]. These epidemiological features have led us to consider *A. braziliense* as the only species causing human CLM, although CLM-like cases have been reported from India, a country where *A. braziliense* is not present [26].

Indeed, *A. tubaeforme* does not penetrate human skin or has a little skin penetration and, although *A. caninum* and *U. stenocephala* are indicated as cause of CLM, their role as agents of skin lesions in humans is still unclear [26,36,116,118,119]. There is an old report of a self-infection by larvae of *U. stenocephala*, which showed that they can penetrate human skin [120]. Skin penetration by larvae of *A. caninum* has been associated with follicolitis, ephemeral and papular/pustolar eruptions [121-123] and to the penetration of muscle fibers and lung infiltrates [124]. However, this latter identification was grasped on epidemiological and biological bases and not on a specific identification of the parasite [124]. Indeed, myositis occurred in human volunteers after skin infection by larvae of this canine hookworm [125], thus corroborating the hypothesis that larval *A. caninum* may indeed cause muscular damage. These larvae have also been associated with a sort of human OLM, a unilateral sub-acute neuroretinitis with loss of vision [126] and, as those of *A. ceylanicum*, can reach adulthood in the human gut. In particular, a relatively newly discovered human disease caused by *A. caninum* is an eosinophilic enteritis regarded as an emergent disease in some areas, e.g. Australia and USA. This syndrome, not known before the 1990’s [126-128], poses important diagnostic challenges. It can be even caused by a single hookworm in the intestinal lumen and is characterized by abdominal pain, discomfort and distension, weight loss, diarrhoea and rectal bleeding [22,128-130]. Occasionally also *A. ceylanicum* can develop to adult stages in human bowel, causing intestinal distress [22,131].

### Treatment and control methods: Need for compromises?

 Different parasiticide classes are available for treatment and control of intestinal nematodes, being (pro-) benzimidazoles (e.g. febantel, fenbendazole), tetrahydropirimidines (e.g. pyrantel), cyclooctadepsipeptides (i.e. emodepside) and macrocyclic lactones (e.g. ivermectin, selamectin, moxidectin, milbemycin oxime) the most used.

Provided below are some key examples of major molecules available for treatment and control of ascarids and ancylostomatids.

A comparative study evaluated the efficacy of three formulations containing mebendazole or fenbendazole alone, or febantel in association with pyrantel and with the cestocide praziquantel [132]. All formulations proved to be effective against infections by ascarids and ancylostomatids in dogs, with different therapeutic efficacies, up to 100% [132]. A multi-centric investigation indicated that the combination of febantel, pyrantel and praziquantel has an efficacy of ~99.9% against canine *T. canis* and hookworms [133]. Another recent study has demonstrated the efficacy and safety of tablets containing pyrantel, oxantel, and praziquantel against natural and/or experimental infections by *T. canisA. caninum* and other canine endoparasites [134]. In cats an association containing pyrantel and praziquantel has high efficacy against ascarids and ancylostomatids [133,135].

Recent experimental and field studies have evaluated the cyclooctadepsipeptid emodepside. Emodepside is available in a spot on formulation for cats (containing also praziquantel), which has efficacy up to 100% in treating infection by *T. cati* or *T. leonina* at different stages [136,137]. This formulation also has 100% efficacy against mature *A. tubaeforme* and efficacy of >95% and >97% against L4 and immature adults, respectively [138]. Tablets marketed for dogs containing emodepside (and praziquantel) have been shown to be safe and efficacious >92-99% against natural or experimental infections caused by L3s and/or L4, immature and mature adults of canine *T. canis* and *T. leonina*[139]. Four different laboratory investigations have demonstrated that this association has >95% and >98% efficacy against larval and adult *U. stenocephala* and *A. caninum*, respectively [140]. A multicentre study evaluating the same anthelmintic association showed high efficacy in reducing egg counts (i.e. geometric mean egg counts reduced by 99.9-100%) in dogs infected by *T. canisT. leoninaU. stenocephalaA. caninum*, under field conditions [141]. Emodepside is also present in a newly marketed oral suspension for dogs, also containing the triazinetrione derivative toltrazuril for the simultaneous treatment of coccidiosis. This formulation has shown efficacy of ≥94.7-99.3% and 100% against immature and adult stages of *T. canis* respectively, and of ≥99.5-100% against adults of *A. caninum* and *U. stenocephala*, respectively, originated from natural and experimental infections [142]. A multicentre investigation carried out across Europe has also indicated efficacy of 100% and 99.9% against *T. canis* and Ancylostomatidae based on faecal egg count reduction [143]. This oral suspension for dogs has been also proved to be effective in experimental feline infections by *T. cati* and *A. tubaeforme*[144].

With regard to macrolactones, the efficacy of a chewable formulation containing ivermectin (and pyrantel) against natural or induced roundworms and hookworms in dogs has been documented to range from 90.1% to 99.6% [145,146]. This association is effective also in the treatment of dogs experimentally infected with *A. braziliense*[147]. In cats with mixed infections, an ivermectin-based chewable formulation showed 92.8% and 90.7% efficacy, respectively, against adult stages of *A. braziliense* and *A. tubaeforme*, while the number of eggs per gram of feces decreased 98.1% by 7 days after administration [148].

By 2000’s the endectocide selamectin has demonstrated efficacy and safety against these parasites [149]. For instance, studies in experimental and natural infections have demonstrated the efficacy of topical selamectin against adult *T. canis* and *T. leonina* and in reducing the fecal excretion of *T. canis* eggs in dogs as well [150]. A series of field investigations carried out in the USA and Europe demonstrated the safety and efficacy of the monthly topical administration of the same ML in the treatment of experimentally and naturally acquired ascaridosis and ancylostomosis in cats [151,152].

A spot-on formulation containing the endectocide moxidectin together with the ectoparasiticide imidacloprid has high efficacy against canine intestinal nematodes in mono-specific and mixed infections [153,154]. For example, in the aforementioned multi-centric study [133] this spot on formulation showed 98.8% efficacy against *T. canis* and 99.9% against Ancylostomatidae. The same spot on formulation has 100% efficacy against adult stages of *T. cati*, up to 98.3% efficacy against immature adults and fourth-stage larvae of the same ascarid, and up to 100% efficacy against adult stages of *Ancylostoma* and immature adults and third-stage larvae of *A. tubaeforme*[133,155].

The ML milbemycin oxime also has high efficacy in removal of roundworms and hookworms from naturally infected dogs and cats with patent infections [156,157]. For instance, adults of *A. caninum* and *T. canis* in naturally infected dogs are killed by milbemycin oxime [158,159]. The molecule has been shown to be effective also in experimental ascaridosis of pups [160] and to have a certain degree of activity against canine ancylostomosis [161,162]. In other trials the molecule has been proven to be active against *T. cati*[163] and fourth-stage larvae and adults of *A. tubaeforme* in cats [164]. Milbemycin oxime is available in associations either with lufenuron or praziquantel. In dogs, the oral associations of milbemycin oxime with lufenuron has shown 91.5% efficacy against naturally ascaridosis [165]. In the multicentre field study mentioned earlier the association containing milbemycin oxime and praziquantel has achieved geometric mean egg counts reduced by 99.4%-99.8% in dogs infected by roundworms and *A. caninum*[141]. In cats, this association has efficacy up to 96.5-100% against fourth-stage larvae and adult stages of *T. cati* and of 93.5% against hookworms [135,166].

Furthermore, milbemycin oxime has been recently marketed in a monthly, chewable, tablet for dogs, also containing the insecticide spinosad. This formulation has a 99.3-100% efficacy in treating and controlling intestinal nematodes in naturally and experimentally infected dogs [167,168].

What emerges is that veterinarians have a broad spectrum of parasiticidal formulations that can be selected according to each individual possible *scenario* and owner and animal compliances. For instance, good compliance (i.e. 87.5%) of the owned pets treated with oral tablets containing pyrantel/oxantel/praziquantel has been documented [134] and, analogously, practices across Europe have reported a high acceptance by dogs treated with the oral tablets containing emodepside [141]. The oral suspension containing emodepside and toltrazuril has an acceptance rated as good and medium in 90% and 9% respectively, in dogs treated in the multi-center study mentioned above [143] and high palatability when administered in cats [144]. Flavoured chewable tablets containing milbemycin oxime presented also high acceptance by treated animals, going from 64% to 94.8% directly from the owners’ hand [141,169]. Hence, several oral formulations, due to their tasty flavor, allow a treatment with minimal distress to the animal and the owner.

It is worth noting that the formulation containing moxidectin and imidacloprid has the advantage of the easy-to-apply dermal spot-on administration in parasitized dogs [170]. This is important also in feline clinical practice, given that indocile or feral cats refusing oral formulations can be easily treated with the spot-on containing moxidectin or emodepside [171,172].

The use of antiparasitic molecules should be programmed also according to other factors, related to the nematode biology and their epidemiological features in different regions. Geographical spread of these parasites, their clinical importance, and especially the high resistance of infectious stages in the environment regardless season or climate (e.g. ascarid ova resist to harsh chemicals, broad temperature ranges and several degrees of moisture), suggest careful attention to prevention approaches [13,88]. Puppies have been considered in the past as the main focus for antiparasitic treatments to control ascarids. However, the demonstrations that intestinal nematodes may indeed infect adult pets and that animals which have been vertically infected by *T. canis* are more susceptible to re-infections if compared to naïve dogs [13,18,46] change our perspective in focused control programs. Also, adult cats may be often re-infected by *T. leonina*, especially if they go outside for hunting [17]. These cats are also more susceptible to infection by *A. tubaeforme*, because they can eat contaminated grass or larvae while grooming, or because larvae can penetrate their skin when they are outdoors. Obviously, the same risk to of being infected by *A. caninum* is run by adult dogs when living or walking in contaminated areas.

Regular “de-worming” or “worming”, an imprecise term but common in daily language today [13], is the basis for an effective chemoprophylaxis irrespective the age of the pet.

Taking control of ascarids as the key example, the major sources of infection and contamination are puppies from 3 weeks and 6 months of age and nursing bitches.

Puppies should wormed with safe formulations able to kill the parasites and to reduce egg shedding in the environment. As an example, a recent study has shown the efficacy of two associations, i.e. milbemycin oxime-based (99.9%) and febantel/pyrantel -based (98.5%), in reducing shedding of *Toxocara* eggs [173]. Given that the lactogenic transmission lasts at least 5 weeks *post partum*, treating puppies at two, four, six and eight weeks of age, and then monthly until 6 months of age may suppress shedding of *T. canis* eggs in the whole period of puppy-hood. The need for a frequent parasiticide administration in pups is due to the continued exposition to re-infections, via the milk and the environment, and to the fact that they may already harbor migrating larvae after birth. If a parasiticide is not administered within the 4^th^ week of age, female ascarids may reach the adulthood and become gravid, thus eggs are shed by the pup when it is as young as about 21 days. No transplacental transmission occurs in cats, thus kittens can be subjected to fortnightly treatments by the 3^rd^ week of age. Given that re-infections may occur throughout the suckling period, dams should be treated with their offspring for the first 2–3 months to avoid patent infections in nursing animals [13,16,17,27].

Treatment of pregnant and/or lactating animals is facilitated by the availability on the market of molecules which can be administered safely in different time periods or for the whole pregnancy and/or lactation, for instance pyrantel or milbemycin oxime, or other broad spectrum drugs. On the other hand, treating pregnant dams is questioned, although sometimes advised in some worm control programs [13,174,175]. Prolonged daily administration of fenbendazole can reduce prenatal infection but such regimen is expensive and can suffer from lack of compliance by the owner [13,176]. Less-frequent administration of ML, can be effective in interrupting vertical transmission with different schemes of treatment [13]. Despite the absence of label claims, such an approach could lead to increased compliance of the owners [13].

Owners and veterinarians should always thoroughly follow manufacturer’s indications for each of the selected parasiticides administered to bitches, queens, puppies and kittens.

Other than these general scientific concepts, indications from the US Companion Animal Parasite Council (CAPC) and the European Scientific Counsel Companion Animal Parasites (ESCCAP) should be taken into account. These two organisations have published guidelines for treatment and control of major parasites affecting companion animals [68,177].

Treatment of puppies and kittens at two, four, six and eight weeks of age is suggested by CAPC. Thereafter, animals should be put on monthly preventives as soon as label recommendations allow. Indeed, kittens do not need to be treated for ascarids until 6 weeks of age but, given the risk for hookworm infection, it is suggested they are treated at 2 weeks of age and then placed on the monthly scheme using molecules effective in preventing heartworm infections and having efficacy against roundworms as well. If puppies and kittens are not treated until 6 to 8 weeks of age or later, they should be put on a monthly preventive according to label recommendations, dewormed again in 2 weeks, and then maintained on monthly preventives thereafter [68]. In other words, a lifelong preventative program, using a “monthly-interval” (i.e. parasiticide administration at 4-week intervals, in accordance with the pharmacokinetics of the molecule used) is supported to exclude any risk of infection for the owners [13]. The veterinarian should monitor and evaluate the efficacy of i) initial treatments, ii) monthly control product, and iii) client compliance by 2–4 fecal examinations in the first year and 1–2 examinations per year thereafter [68].

The ESCCAP recently advised that pups should receive a parasiticide at 2 weeks of age, then at fortnightly intervals until two weeks after weaning. Thereafter, puppies should undergo monthly treatments until six months old. Fortnightly treatment of kittens can start at 3 weeks of age and should be repeated fortnightly until two weeks after weaning, then monthly for six months. With regard to adult dogs and cats, annual or twice yearly treatments for *Toxocara* spp. does not reduce the risk of patent infections and, also, worming four times a year does not necessarily eliminate patent infections; conversely, the ESCCAP states that monthly worm treatment can largely prevent patent infections [177]. In other words, a treatment frequency of at least 4 times per year, or at intervals not exceeding 3 months, or even a monthly treatment, are general recommendations, according to different *scenarios*, e.g. real zoonotic risks, presence of children in the pet owners family, pregnancy of bitch or queen, housing conditions [13,177]. When a year-round-control is not performed (e.g. because an owner disagrees with a frequent anthelmintic administration, or local legislation requires diagnosis or risk assessment prior to treat an animal), regular faecal examinations (e.g. every 1–3 months) of susceptible animals is considered a feasible way of evaluating the re-occurrence of intestinal nematodes [177].

A compromise between these two views from North America and Europe seems to be a good choice [13], if particular situations do not apply. A minimum number of 4 administrations per year or treatments at intervals of 4–6 weeks can be effective in preventing most patent infections, while a worming frequency of less than 3–4 times per year does not influence parasite prevalence [13,178]. Nonetheless, no impact on patent parasitic infections in pet populations has been found after annual or bi-annual anthelmintic treatments [51]. In this latter study more than a half of a Swiss canine population has been found to shed helminth eggs at least once in 1 year despite quarterly deworming. More specifically, a yearly incidence of 32% of *T. canis* infection has been found in dogs that received four anthelmintic treatments per year [51].

As mentioned earlier, in US settings the routine monthly parasiticide administration is sometimes performed along with annual or semi-annual fecal examinations [47,68].

In any case, the monthly treatment approach appears to have several benefits, especially when performed with a macrolactone, which can accomplish the suppression of most important parasitic nematodes affecting pets [179].

On the other hand, the possibility of using a year round treatment is particularly important in those regions where there is a systematic necessity to perform the annual chemoprophylaxis for other major parasites. A year-round control program with molecules which can be monthly administered for the prevention of cardio-pulmonary nematodes, i.e. *Dirofilaria immitis* (e.g. ivermectin, moxidectin, milbemycin oxime) and *Angiostrongylus vasorum* (i.e. moxidectin), is powerful also to achieve a decrease in prevalence of intestinal nematodes [49,180].

A year round program would be powerful also in feline patients, given that the level of nematode transmission is higher in free-roaming cats than in cats which receive adequate sanitary care [181]. However, recent studies have proved high prevalence rates, at least for *T. cati*, also in pet household cats [182-184].

The other side of the coin says that frequent use of anthelmintics in companion animals could have detrimental effects. In the past decades the abuse of parasiticides has led to the emergence of livestock and horse parasites resistant to one or more anthelmintic classes. As a general approach, the administration of a broad-spectrum parasiticide without a copromicroscopic examination should be discouraged considering that the unnecessary use of anthelmintics has major influence in promoting drug resistance. Hence, a concern related to frequent anthelmintic treatments could be an increase of drug resistant populations of pet nematodes, especially for long-term indiscriminate use of parasiticides, which have been on the market (often over-the-counter) over a long time. Indeed, at the moment there is only evidence of resistance to pyrantel in *A. caninum*[185]. Although pyrantel is not used for monthly prevention of cardio-pulmonary parasites and no data have emerged for roundworms, a high level of attention should always be maintained to detect any hint of drug resistance in pet nematodes. This is even more relevant considering that there is the first laboratory evidence that *D. immitis* can develop a degree, although yet to be established, of survival to certain parasiticides [186].

The present very limited evidence of drug resistance in small animal parasites is likely due to the fact that pets are most often kept individually or in small numbers, thus dynamics of parasitic populations and influence on *refugia* is very different from what happens in livestock. Pets are usually treated individually, thus most roundworms and hookworms in a given area escape from the treatment and remain in the *refugium*[13]. Nonetheless, the indiscriminate use of anthelmintics in concentrated groups of small animals (e.g. kennels, colonies, shelters, breeding facilities) might nurture the development of resistant nematodes [187].

### The importance of copromicroscopic examinations

The misconceptions that only young animals should be dewormed and a single treatment clears a “generally” parasitized animal, induce negligence in performing diagnostic copromicroscopy in veterinary practices.

Conversely, systematic copromicroscopic examinations should be regularly instituted for companion animals, which, in turn, are virtually subjected for all their lifespan to continuous re-infection by roundworms and hookworms, even when they have a lifestyle far from that of stray animals or of animals kept in shelter or refuges [47,188-190].

Copromicroscopic techniques, e.g. floatation methods or commercial kits, are easy to perform in clinical practices. Nonetheless, diagnostic challenges may arise for both ascaridosis and ancylostomosis. Pre-patency period greatly affects diagnosis and effective control programs: parasitic ova can be detected at faecal examination only after nematode development, mating and patency, i.e. in some cases even a few weeks post infection and appearance of clinical signs. As a key example, it has been shown that the vast majority of dogs aged less that 6 weeks are infected by intestinal roundworms, although they may score negative at the copromicroscopic examination [6,191]. The same challenge may occur in puppies infected by hookworm larvae originated from their dam, in that these nematodes shed eggs by the tenth day of infection, thus after the symptoms appear. Diagnosis in these pups may be achieved only on clinical signs like, for instance, pale mucosae and soft to liquid dark faeces. Also, symptoms caused by acute ancylostomosis due to sudden exposure to infective larvae in whelps and adults may appear about four days before egg shedding, thus making diagnosis very problematic [16,27].

Faecal examinations should not be related to patient’s health and must be performed regardless of the presence of gastrointestinal symptoms (e.g. diarrhoea, vomiting, etc.). This is of importance given that, for instance, no significant differences in nematode infection in symptomatic dogs, compared with animals without clinical signs has been found [49,53,192]. However, asymptomatic animals are usually considered parasite-free, thus their owners may be not interested in routine examination for parasites with subsequent treatment if necessary [59]. Such an approach should be discouraged, given that asymptomatic dogs are as likely to be infected as animals with clinical evidence. Therefore, these categories present the same level of zoonotic risk, which may not be fully appreciated by pet owners [59,193-195]. There are, however, situations where awareness of pet owners on zoonotic diseases is very high and they accept to have their pets undertaken on regular control plans [47,196]. Furthermore, repeated faecal examinations throughout the lifespan of a pet are of paramount importance even in well- cared for dogs and cats, given that recurrence of parasites is possible, regardless of whether they undergo a control anthelmintic program or not [51,53].

**Figure 3:**
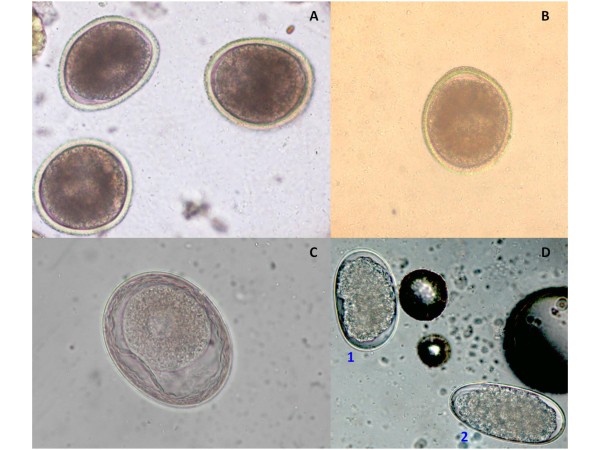
**Floatation with zinc sulphate: eggs of*Toxocara canis*(A),*Toxocara cati*(B),*Toxascaris leonina*(C),*Uncinaria stenocephala*(D1) and*Ancylostoma caninum*(D2).**

From a practical standpoint, the pneumonic phase of larval migrations can only be suspected for the simultaneous appearance of respiratory symptoms in all puppies of a litter within two weeks after birth. Specific diagnosis of patent toxocarosis is achieved through standard copromicroscopic floatation because eggs of *T. canisT. cati* and *T. leonina* (Figure 3) are usually present in high number and easy to identify. However, in certain areas of the World (e.g. North America) the raccoon roundworm *Baylisascaris procyonis* can pose diagnostic challenges. This ascarid causes patent infections also in dogs [197,198], and its eggs greatly resemble those of *T. canis*, thus representing a diagnostic problem and an important danger for human health. In fact, the vast majority of humans who ingest infective eggs of *B. procyonis* suffer from severe permanent neurological damage or even die [199]. Hence, at least where this parasite is endemic, veterinary personnel must be skillful and trained in recognizing eggs of *B. procyonis* for preservation of the public health of people eventually exposed to faeces eliminated by dogs infected by *B. procyonis*. Even though this is true for North America, this parasite has been introduced also in Europe and Asia with its natural wild host [200]. Hence, what the future will hold on this life-threatening zoonosis in other continents is currently unknown.

Diagnosis of ancylostomosis in dogs and cats cannot be achieved at the species level for the overlapping morphological and morphometric features of *Ancylostoma* spp. eggs (Figure 3). Coprocultures can be performed for a specific diagnosis but, from a practical standpoint, the presence of hookworm eggs in pets’ faeces would require a parasiticide treatment regardless of the species affecting the animal. Eggs of *U. stenocephala* (Figure 3) can be identified by their larger size [36].

Veterinarians should convince pet owners of the importance of periodic faecal examinations. In the first year of life any pet should undergo at least 2–4 copromicroscopic examinations and then, when adult, it should be examined more than once per year according to health status, lifestyle and frequency of treatments. Specifically, free ranging animals and those living indoors but allowed to go outside should always be subjected to regular examination of the faeces. Particular attention should be given to copromicroscopic analyses carried out in the worm control programs. When a monthly-based treatment program is not performed, faecal examinations every 1–3 months of susceptible pets is an efficacious way of evaluating the re-occurrence of intestinal parasitoses in previously treated animals. Indeed, post-treatment faecal examinations are important to evaluate success of drug administration and, in a future perspective, to detect any indication of drug resistance in ancylostomatids and ascarids. On the other hand, coprophagy is relatively common in dogs, thus the presence of eggs in stool samples after treatment could often be due not to failure of treatments but rather to ingestion of their own defecation or other animals’ faeces [84].

### Awareness of the general public and pet owners: A need to enhance risk perception without causing alarm

Controlling ascaridosis and ancylostomosis in pets is crucial to reduce infection risk for other companion animals and to minimize public health hazards. All categories involved in pet medicine should take care of animal health and public behavior, given that human syndromes caused by pet nematodes may lead to permanent damage [18,88].

Awareness of pet owners and the general public and continuous education of veterinarians are at the basis of effective prevention. Dissemination of understanding and knowledge of transmission routes, at-risk categories and areas, and control methods are pivotal to minimize possibilities of human and animal infections. Although virtually all of the majority of roundworms and hookworms affecting dogs and cats may cause human diseases, the public risk perception in general is poor. As a key example, despite the infection by *Toxocara* spp. is the most prevalent human helminthosis in some industrialized countries, public awareness of this syndrome is scant [201]. A survey carried out in the UK has shown than less than the half of the people interviewed, including pet-owners and non-pet-owners, perceived the risk of transmission of nematodes from pets to humans. Interestingly, no differences in hazard awareness was found between people who owned a pet and people who did not [202]. Another study performed in a developed area of Brazil has demonstrated that the majority of dog owners did not know about intestinal parasites, sources of infections, possible risk factors for zoonoses and focused prophylactic measures. Therefore, the high presence of zoonotic species in owned dogs of the studied region, along with the lack of information known by owners, endorse that the risk of zoonotic infection by canine intestinal nematodes may be indeed high [59].

Veterinarians, who are the most appropriate and questioned source of information on zoonoses, should always provide pet owners with accurate worming schedules and their efficacy, and appropriate all-day-life measures. This is of particular importance if one considers that veterinarians are a more congruous source of knowledge on zoonoses than physicians [203]. Nonetheless, lack of education has been well documented also in veterinary professions [203-205]. In Canada less than the half of practitioners working in small animal practices declared that they talked with pet-owners about the zoonotic risk of pet endoparasites, while the remaining did so only under particular circumstances or did not at all [206]. In the USA it has been recently suggested that a national surveillance program should be established in order to better understand specific aetiology of human *larva migrans* syndromes and their actual incidence, in order to aim for focused intervention programs [18]. More interview- based studies are warranted in other countries to implement our understanding of how the risk perception is diffused in the population and, more importantly, to implement awareness of the general public and of pet owners.

**Figure 4:**
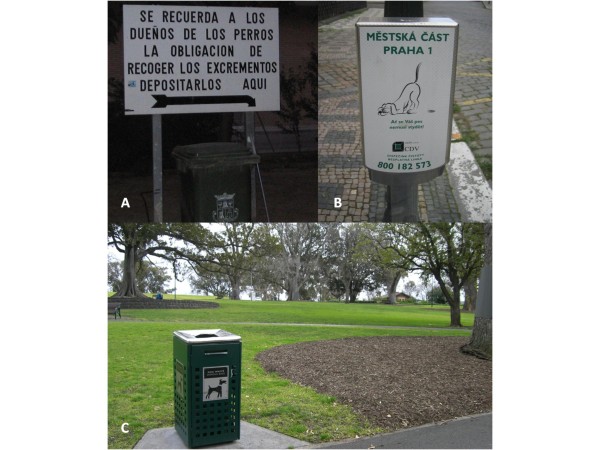
Dog-reserved areas and indications for pet owners in public areas in Valencia, Spain (A), Praha, Czech Republic (B) and Melbourne, Australia (C).

No practical methods exist to eliminate infective elements of intestinal nematodes of pets (especially for ascarids) present on the ground, thus prevention of initial contamination of the environments is the key goal. Veterinarians should inform all owners about ways of transmission for their pet and for themselves, and about clinical evidence for diseases and methods of prevention. On the other hand, any pet owner should clearly acquire relevant information and have appropriate behavior. In fact, an important cause of the heavy outdoor presence of infectious parasitic elements is lack of education (and of awareness on the actual zoonotic risk) by pet owners. Sites shared by children and animals like backyards, sandpits, parks, playgrounds, beaches, often represent a risk for the heavy contamination by pet faeces. Public parks may be highly contaminated by eggs of *T. canis*, while sand-boxes and sand-pits by those of *T. cati*[18,88,90,207,208]. Hence, common prevention practices, which should be known by any pet owner are covering sandboxes, avoiding animal defecation in public areas or at least always clean animal faeces from the ground. In fact, educating owners on regular removal and disposal of faeces and to empty cat litter trays is of paramount importance to minimize environmental contamination and risk of transmission to both animals and humans [6,79]. When walking their pets in public areas, all owners should respect local indications (Figure 4) and keep their animals in reserved areas, if present.

With regard to hookworms, sites favoring survival and development of free-living larvae are shaded, warm, humid and well-drained soils. Furthermore, unpaved runs are highly favorable for hookworm larvae because of mixing of faeces and soils. Prevention of animal and human ancylostomosis relies on adequate hygiene measures (e.g. hand and feet washing), removal of pet faeces and, importantly, wearing shoes and avoiding lying on risky areas or, in any case, where there is the likelihood that animals usually defecate.

### Hookworms and travelling

Tourists travelling in at risk zones for CLM should be careful in walking barefoot on beaches and in lying down on the sand, especially because *A. braziliense* is endemic in popular tourist areas. These human categories are susceptible to infection at their vacation destination and then may return home infected at the end of the holiday, often providing a challenging dilemma to their physician.

Nonetheless, autochthonous reports of CLM have been reported from countries where the tropical *A. braziliense* is absent, thus these cases are considered “unexpected” [26]. Unfortunately no identification to species of larvae found at biopsies has been performed, thus only hypotheses are available on the identity of hookworm involved. As key examples, CLM cases possibly by *U. stenocephala* and/or *A. caninum* have been recorded in the UK and New Zealand. Several other local cases of CLM by unidentified hookworms have been described in Italy, Serbia, Germany, France and UK as well [26]. Although few in number, these cases in areas where *A. caninumA. tubaeforme* and *U. stenocephala* are present, show a possible risk for human infections, characterized by skin conditions, which, possibly, could also involve other conditions (e.g. pneumonitis). Also, it cannot be ruled out that these few cases published could be the tip of the iceberg and that others are not diagnosed or not even referred to physicians. The number of CLM in “unexpected” countries could be much higher than thought, thus not only travelers and tourists spending holidays in tropical zones considered “risky” can be faced with these infections but all humans exposed to soil contaminated by larval hookworms.

## Conclusion

**Figure 5:**
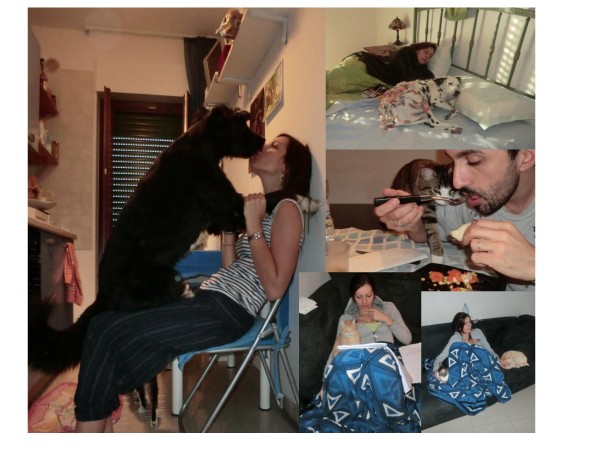
**Close contact between privately owned pets and their owners. **Although this behavior can be questionable, it cannot be considered at risk of infection with zoonotic nematodes for the owners.

Given the clinical importance of intestinal nematodes affecting pets, their ubiquitous presence and the zoonotic impact some of them have, public education is crucial for reducing risk exposure in both humans and companion animals. At the same time pet owners and, in general, the public opinion should maintain a self-confidence that keeping a pet is safe and a positive experience. This is also true when close-contact occurs between the pet and the owner, even when some behaviour can be questionable (Figure 5). Actually, it is established that direct contact with infected pets presents no relevant risk in the transmission of intestinal nematodes and there is no association between pet ownership and infection occurrence [78,79]. Owners should have confidence that ownership of any companion animal is beneficial and safe as long as their pets are healthy [18]. Pet owners enjoy a plethora of advantages by living with dogs and/or cats. For instance, children, the elderly and disabled persons particularly benefit from their contact with a beloved pet. Companion animals represent a way of life for a lot of the people and this relationship provides socialization, mental health, and physical well-being: those who own a pet have been shown to display reduction in blood pressure and cholesterol levels, require less medical care, and it has also been reported that there is an improvement of life quality and quick recovery after heartbreaking events [209-213]. Therefore, owning companion animals is vital for the majority of families, especially when children and the elderly are present [212,214]. However, the potential risks of pet-originating zoonoses should always be kept in mind. This has become even more so in recent years, when several sociological changes have influenced the relationships between physicians and veterinarians. In fact, the major goal of the re-discovered “One Health Program” (i.e. “*the collaborative work of multiple disciplines to help attain optimal health of people, animals, and our environment*”) highlights the crucial role of a tight tie between the human health operators, vet practitioners, and the general public [213].

For instance advances in chemotherapy for AIDS and new possibilities of organ transplants, and the prolonged life expectancy, have increased the number of immunocompromised patients in human communities. These subjects need to be aware of possible zoonotic parasites and of all measures to prevent infections for their pets and for themselves. It is worth highlighting that immunocompromised individuals should not give up their animal, as it has been demonstrated that pet ownership minimizes depression and that standard hygiene measures reduce at minimum the risk of acquiring zoonotic infections in this particular category [215,216].

A desirable goal for effective control programs would also be to understand which are changes and trends in terms of prevalence of infection by ascarids and hookworms in canine and feline populations, with the simultaneous aim to track incidence of human cases caused by each of the single aetiological agents. This would be a basic step to cope with current weaknesses in prevention approaches and to establish where to intervene with focused plans. In fact, updated information on prevalence of parasites of dogs and cats and the risk factors associated with infection, as well as reinforcing veterinary and public health concerns, is of crucial relevance because common awareness is non-existent or often based on outdated information.

The cornerstone to control intestinal parasitoses of pets is a combination of strategic worming methods (especially puppies, kittens and dams), wearing footwear when needed, supervising playing children and their interactions with pets, breaking faecal-oral routes by washing hands and removal and disposal of faeces from public and private grounds and litter trays, alimentary habits [216-218].

Ten years ago it was perceived that veterinary parasitology was becoming irrelevant in routine clinical practice [219]. Regrettably, after more than ten years this perception is practically a reality in several settings. The involvement of practitioners in a worming control program is no more than the administration of one of the several broad-spectrum parasiticides available on the market, even in the absence of evident parasitosis or without a copromicroscopic examination. Such a fallacy comes from the misconceptions that a deep knowledge of epidemiology and biology of certain parasites is superfluous and that control of major helminths can be achieved just with a periodic medication. Nonetheless, roundworms and hookworms remain today the most diffused nematodes affecting pets around the world and they still cause zoonotic infections in humans. There is, therefore, the evidence, that scientists, pet owners and veterinarians should re-consider their approach on parasitology and foster their interest not only in emergent parasites like cardio-pulmonary nematodes or water-borne protozoa, but also in “old-fashioned” intestinal worms.

Given that parasitic zoonoses are too often neglected or underappreciated, and may be mismanaged or underdiagnosed by both veterinarians and physicians [213], a strong education outreach by veterinary and medical practitioners should be accomplished [214,220]. Veterinarians must keep their guard up against zoonotic parasitoses of pets and constantly provide advice and improve knowledge of their clients, with a special focus on those human categories, who are at higher risk of infection, in order to allow pets to remain integral members of household and families. Furthermore, owners should become aware of “invisible” beneficial effects of a lifespan control program based on routine faecal examinations and frequent worming.

New concepts for accurate preventative plans have been generated based on several individual and epidemiological circumstances. The role of the veterinarians and constant compliance of the owners are crucial for the success of worm control programs in pets. Additionally, the present climate changes and global warming supports the need for a continuous global worming, given that faster egg embryonation and increased over-wintering of infectious elements in the environment will likely increase the spread of helminths affecting companion animals and humans in several areas of the World, as recently hypothesized for sub-Arctic and Arctic regions [221].

## Competing interests

Scientific aspects of the article have not been influenced by any third party.

## Author contribution

DT conceived the intellectual content of the article and wrote the text.
